# Cyclophilin D is a new non-canonical substrate of the mitochondrial intermembrane space assembly pathway

**DOI:** 10.1016/j.jbc.2025.110883

**Published:** 2025-11-04

**Authors:** Mara Equisoain Redin, Veronica Bazzani, Eve Harding, Joshua McHale, Carlo Vascotto

**Affiliations:** 1 IMol Polish Academy of Sciences, Warsaw, Poland; 2 Department of Medicine, University of Udine, Udine, Italy

**Keywords:** cyclophilin D, Mia40, mitochondria, protein import, redox

## Abstract

Mitochondrial protein import is essential for organelle function and cellular homeostasis. While Cyclophilin D (CypD) is a well-characterized regulator of the mitochondrial permeability transition pore (MPTP) and resides in the matrix, the mechanisms underlying its import remain poorly defined. In this study, we identify CypD as a novel non-canonical substrate of the mitochondrial intermembrane space assembly (MIA) pathway mediated by the oxidoreductase Mia40. Structural analysis revealed conserved cysteine pairs in CypD that are compatible with disulfide bond formation. Using *in vitro* pull-down assays, we demonstrate a redox-sensitive interaction between CypD and Mia40, which was further confirmed by co-immunoprecipitation and proximity ligation assays. Expression of CypD cysteine mutants in cells revealed that residues Cys82 and Cys203 are critical for Mia40-dependent interaction and protein stability. Notably, expression of the Cys203Ala mutant significantly reduced cell viability, suggesting a key functional role for this residue. Functional experiments showed that depletion of Mia40 leads to a significant reduction in mitochondrial CypD levels, a result that was confirmed in a series of leukemia cell lines with variable Mia40 expression. Our results shed light on a previously unrecognized import mechanism for CypD and expand the known substrate repertoire of Mia40, demonstrating that the MIA pathway also contributes to the import of mitochondrial matrix proteins. This work highlights the functional versatility of the MIA pathway beyond the intermembrane space and reveals an additional regulatory level in mitochondrial proteostasis with implications for cell death signaling and mitochondrial pathophysiology.

Mitochondria are dynamic, multifunctional organelles essential for cellular energy production, metabolism, and stress responses. While best known for their role in ATP generation *via* oxidative phosphorylation (OXPHOS), mitochondria also play key roles in calcium homeostasis, reactive oxygen species (ROS) signaling, programmed cell death, and the biosynthesis of critical metabolites, such as heme and steroid hormones (1, 2). Their functional complexity depends on the coordinated expression of both mitochondrial and nuclear genomes. Notably, more than 99% of mitochondrial proteins are encoded by nuclear DNA and imported post-translationally into the organelle (3). These proteins are sorted into the outer membrane (OM), intermembrane space (IMS), inner membrane (IM), or matrix *via* tightly regulated import pathways that ensure proteostasis and subcompartmental specificity (4, 5).

Cyclophilin D (CypD) is a mitochondrial peptidyl-prolyl cis-trans isomerase (PPIase) that localizes to the matrix and acts as a key regulator of the mitochondrial permeability transition pore (MPTP), a high-conductance channel implicated in both necrotic and apoptotic cell death under stress conditions (6, 7). CypD-mediated MPTP opening leads to the collapse of mitochondrial membrane potential, inhibition of ATP synthesis, and release of pro-apoptotic factors, linking mitochondrial dysfunction with cell fate decisions (8). CypD is encoded by the nuclear *PPIF* gene and is synthesized as a 22-kDa precursor bearing an N-terminal mitochondrial targeting sequence (MTS), which is cleaved upon translocation into the matrix to yield a mature ∼19-kDa form (9, 10). Despite its well-characterized mitochondrial localization, the molecular mechanisms underlying CypD import remain unknown.

The mitochondrial intermembrane space assembly (MIA) pathway is a redox-regulated system that mediates the oxidative folding and import of cysteine-rich proteins into the IMS. This pathway relies on the oxidoreductase Mia40, which forms transient intermolecular disulfide bonds with substrate proteins, facilitating their oxidation and proper folding (11). Mia40 typically recognizes substrates containing conserved cysteine motifs, particularly twin CX3C or CX9C motifs, and catalyzes the formation of intramolecular disulfide bridges that promote retention and functionality in the IMS (12, 13). Canonical Mia40 substrates are structurally conserved proteins of approximately 10 to 15 kDa, such as small TIM chaperones and cytochrome *c* oxidase assembly factors. Mia40 functions in concert with the sulfhydryl oxidase ALR, which reoxidizes Mia40 to sustain its catalytic activity (13). However, accumulating evidence has expanded the known substrate repertoire of Mia40 to include non-canonical proteins that deviate from classical cysteine motif patterns, exhibit atypical structures, or exceed the typical size range of IMS proteins (14). These include factors involved in mitochondrial DNA repair, translation, and apoptosis, some of which are partially or entirely localized outside the IMS. For instance, the DNA repair enzyme APE1 requires Mia40 for mitochondrial import and oxidative activation, despite its primary function in the matrix (15). Similarly, MICU1, a regulator of mitochondrial calcium uptake, has been identified as a Mia40 substrate, although it partially localizes to the inner membrane (16). These findings challenge the traditional view that Mia40 acts exclusively within the IMS and suggest broader physiological roles for this redox-regulated import machinery.

Analysis of the crystallographic structure of CypD reveals two pairs of cysteine residues (Cys82–Cys203 and Cys104–Cys157) whose alpha-carbon distances fall within the range compatible with disulfide bond formation (17). This structural feature raised the hypothesis that CypD may be recognized and oxidatively modified by Mia40 during mitochondrial import. In this study, we provide multiple lines of evidence that support the Mia40-dependent import of CypD into the mitochondria. These findings confirm and extend the involvement of Mia40 in the import of a mitochondrial matrix protein and identify CypD as a novel non-canonical substrate of the MIA pathway. This work not only expands the substrate repertoire of Mia40 but also highlights the complexity of mitochondrial protein import mechanisms and their intersection with redox biology.

## Results

### CypD localizes in the mitochondria compartment and its translocation is independent of the mitochondrial membrane potential

**Figure 1:**
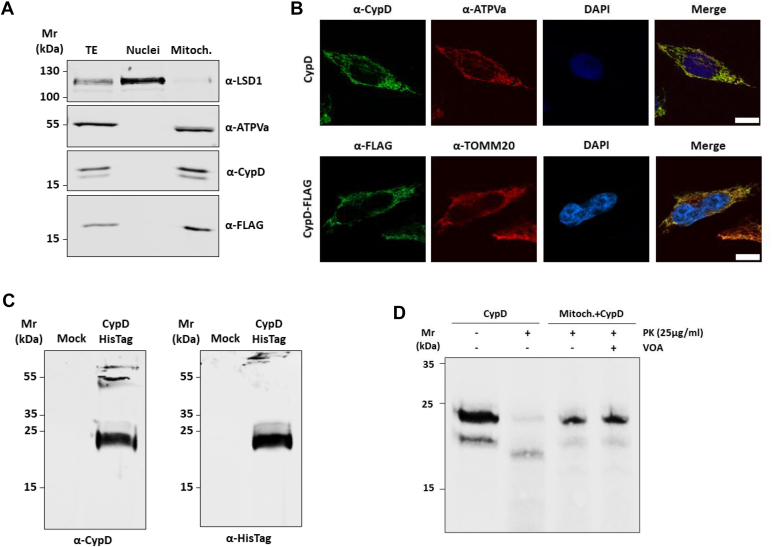
**CypD localizes in mitochondria.***A*, Western Blot analysis of total (TE), nuclear (Nuclei), and mitochondrial (Mitoch.) protein extracts of HeLa cells transiently expressing FLAG-tagged CypD. Endogenous and ectopic CypD are present only in the mitochondrial protein extract. ATPVa and LSD1 were used as mitochondrial and nuclear marker, respectively. *B*, confocal immunofluorescence analysis showing endogenous CypD (CypD) and ectopic (CypD-FLAG) localization in HeLa cells. 12 h after transfection cells were fixed and immunofluorescence was carried out with anti-CypD and anti-FLAG antibodies as reported in material and method section. ATPVa and TOMM20 were used to stain mitochondria. Mitochondrial localization of both endogenous and ectopic CypD is visible in merged image as yellow signal. Magnification: 63x; scale bar represents 10 μm. *C*, Western blot analysis of *in vitro* synthesized recombinant CypD-HisTag. The protein was expressed in Wheat germ system, separated in 8M urea denaturing gel, immunoblotted, and analyzed with and anti-CypD (*left*) anti-HisTag (*right*) antibodies. A single band at the expected molecular weight accounting for the full-length form of CypD is visible. *D*, *in organello* import analysis of recombinant CypD expressed *in vitro* with Met^35S^. Incubation of precursor protein with mitochondria isolated from HEK-293 protect CypD from protease K (PK) digestion. Loss of membrane potential by VOA treatment (valinomycin, oligomycin, and antimycin A) did not affect the internalization of CypD. Representative image of four replicates.

To confirm the mitochondrial localization of CypD, the cDNA of the full-length protein was cloned into the eukaryotic expression vector pCMV5.1-FLAG and transfected into HeLa cells. Total (TE), nuclear (Nuclei), and mitochondrial (Mitoch) protein extracts were separated on SDS-PAGE, immunoblotted and analyzed for the presence of both endogenous CypD and ectopically expressed FLAG-tagged CypD (Fig. 1*A*). According to the data reported in literature, both endogenous and ectopically expressed CypD were detected only in the mitochondrial fraction and were completely excluded by the nuclear fraction, confirming the exclusive mitochondrial subcellular localization of CypD. To further support this data, immunofluorescence studies were performed. As shown in Figure 1*B*, both endogenous and ectopically expressed CypD colocalize with the mitochondrial markers ATPVa and TOMM20, confirming their strict mitochondrial localization. Finally, to verify if the mitochondrial translocation of CypD required an active mitochondrial membrane potential (MMP), *in vitro* expressed CypD was used to perform *in organello* import studies. Radiolabeled full-length CypD, incorporating [^35^S]-methionine and tagged with a C-terminal 6x-HisTag was expressed using the Wheat germ system. Then, 2 μl of recombinant protein were separated on a urea gel, immunoblotted and recognized with anti-CypD and anti-HisTag antibodies to confirm the correct expression of the protein (Fig. 1*C*). As expected, a single band with a gel mobility of approximately 22 kDa, compatible with the molecular weight of full-length CypD, was detected. In parallel, the same protein was used as a precursor in the *in organello* import assay (Fig. 1*D*). Treatment with proteinase K (PK) completely degraded the precursor protein (Lane 2), while upon 30 min incubation with isolated intact mitochondria from HEK-293 cells, CypD entered the mitochondria, resulting in protection from degradation (Lane 3). Notably, disruption of the MMP, treating mitochondria with a combination of valinomycin, oligomycin, and antimycin A (VOA), did not prevent the internalization of the precursor (Lane 4), proving that CypD translocation is voltage independent.

### CypD interacts with Mia40

**Figure 2:**
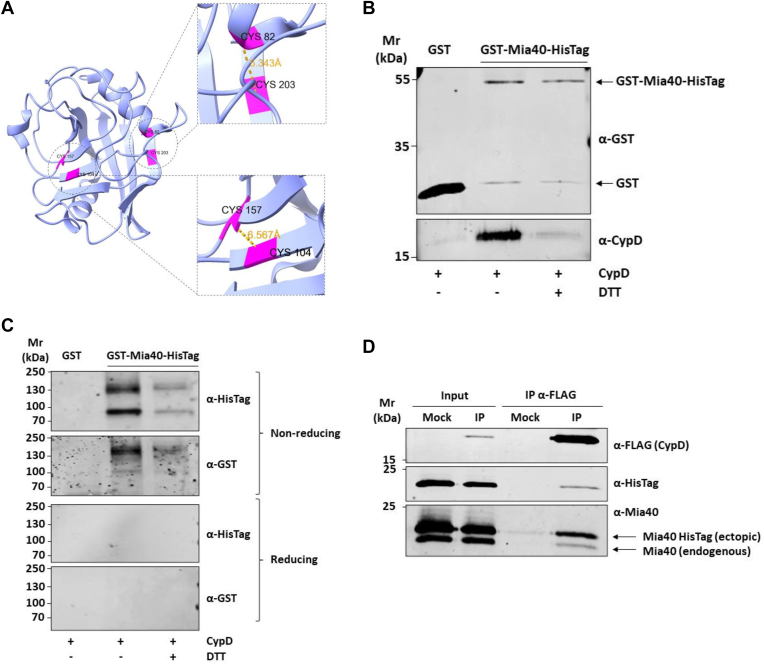

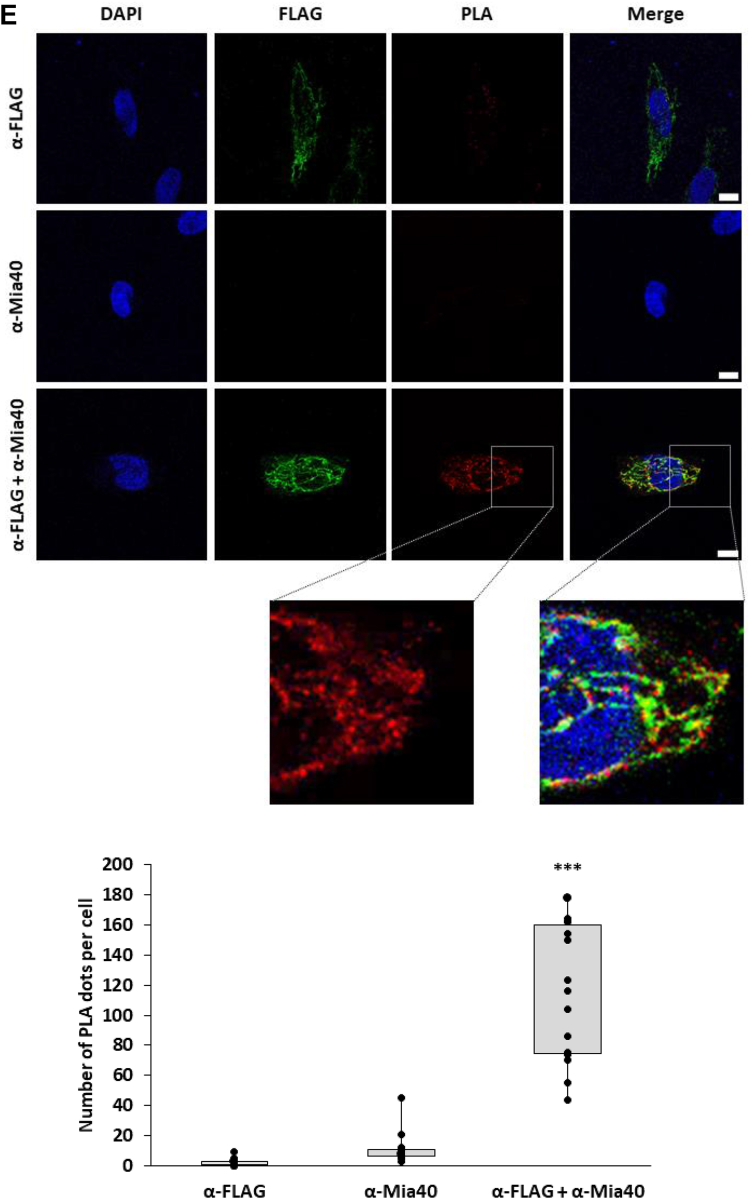
**CypD interacts with Mia40 *via* disulfide bridge formation.***A*, Structure analysis of Human CypD. In *pink* are shown adjacent cysteine residues and in *yellow* the distance among adjacent alpha carbons compatible with the formation of disulfide bridges. *B*, Western blot analysis of GST pull-down assay. Recombinant GST-Mia40-HisTag protein was used as bait, GST alone as control, and purified CypD-HisTag as prey. Interaction between the two proteins was disrupted after treatment with the reducing agent DTT. *C*, Western blot analysis of GST pull-down assay separated on SDS-PAGE gel under non-reducing and reducing conditions. Under non-reducing conditions low-mobility bands immunoreactive for anti-CypD and anti-GST are visible accounting for a stable complex formed by CypD and Mia40 (*upper panel*). Interactions are disrupted by DTT treatment and no longer visible by separating samples in reducing conditions (*lower panel*). *D*, Western blot analysis of affinity purification from HeLa cells expressing CypD-FLAG and Mia40-HisTag proteins. HeLa cells were transiently transfected with pCMV5.1 (Mock) and pCMV5.1-CypD-FLAG vectors. Control (Mock) and CypD-FLAG (IP) were immunoprecipitated under native conditions from total HeLa cell extracts, separated by 12% SDS-PAGE, and analyzed by Western blot. Both endogenous Mia40 and recombinant Mia40-hisTag were enriched in the IP fraction. *E*, representative immunofluorescence images (*upper panel*) and graph (*lower panel*) of PLA analysis between ectopic CypD-FLAG and endogenous Mia40. HeLa cells were transiently transfected with pCMV5.1-CypD-FLAG vector. CypD expression was detected by using an anti-FLAG antibody (*green*), while PLA signal are visible as red dots. In the control transfected with pCMV5.1-empty vector and the control transfected with pCMV5.1-CypD using only anti-FLAG or anti-Mia40 antibody, no PLA signals are detected. White bars corresponded to 10 μm. Data reported in the histogram accounted for the average number of PLA dots of at least 15 randomly selected cells per condition (∗∗∗: *p* ≤ 0.001).

Once confirmed that our experimental settings can reproduce the mitochondrial localization of CypD, we focused on the molecular mechanisms characterising the translocation of CypD. By analyzing the crystallographic 3D structure of CypD, we observed the presence of two adjacent pairs of cysteine residues, Cys104-Cys157 and Cys82-Cys203, with distances between the alpha carbons measuring 6.567 Å and 5.343 Å, respectively (Fig. 2*A*). The distance between the alpha carbon atoms of cysteine residues involved in a disulfide bond can range from 3.0 Å to 7.5 Å (18), suggesting the possibility that CypD is a non-canonical substrate of the MIA pathway.

To verify this hypothesis, a GST pull-down assay was conducted using GST-Mia40-HisTag as ‘bait’ and *in vitro* synthesized and purified recombinant CypD-HisTag as ‘prey’ (Fig. S1). GST protein alone was used as a negative control (Fig. 2*B*). As shown in Figure 2*B*, CypD strongly interacts with Mia40 (Lane 2). Incubation of the reaction with the reducing agent dithiothreitol (DTT) led to an almost complete loss of CypD signal in the pull-down fraction (Lane 3) confirming that the binding between the two proteins occurs through the formation of a disulfide bridge. To confirm the formation of a Mia40/CypD complex, GST pull-down samples were also separated under non-reducing conditions and immunodetection was performed with anti-CypD and anti-GST antibodies (Fig. 2*C*). In both cases, high-molecular weight bands were visible accounting for the presence of a stable complex between the two proteins (Lane 2). These bands were not present in samples treated with DTT (Lane 3) or by separating the samples under reducing conditions. Having proved *in vitro* the interaction between Mia40 and CypD, we next evaluated if this binding was also occurring *in vivo*. HeLa cells were transiently transfected to express Mia40-HisTag and CypD-FLAG (IP), or the empty vector as control (Mock). Then, CypD-FLAG was immunopurified and Mia40-HisTag protein was detected in the IP fraction. Remarkably, not only the ectopic His-tagged protein but also the endogenous Mia40 was detected in the IP fraction (Fig. 2*D*).

To further demonstrate the interaction between CypD and Mia40, we performed a proximity ligation assay (PLA) between the ectopic CypD-FLAG and the endogenous Mia40. The detection of PLA signal confirms that the CypD interacts with Mia40 (Fig. 2*E*). As reported in the graph of Figure 2*E*, dots are present only in the reaction with both antibodies (*lower panel*), while in the control reactions omitting the anti-Mia40 (*upper panel*), or the anti-FLAG (*central panel*) the PLA signal is almost completely absent, proving the specificity of the assay. Altogether, these results support the hypothesis that CypD is a new non-canonical substrate of the MIA pathway.

### Identification of critical cysteine residues in CypD that may be involved in the disulfide relay system

**Figure 3:**
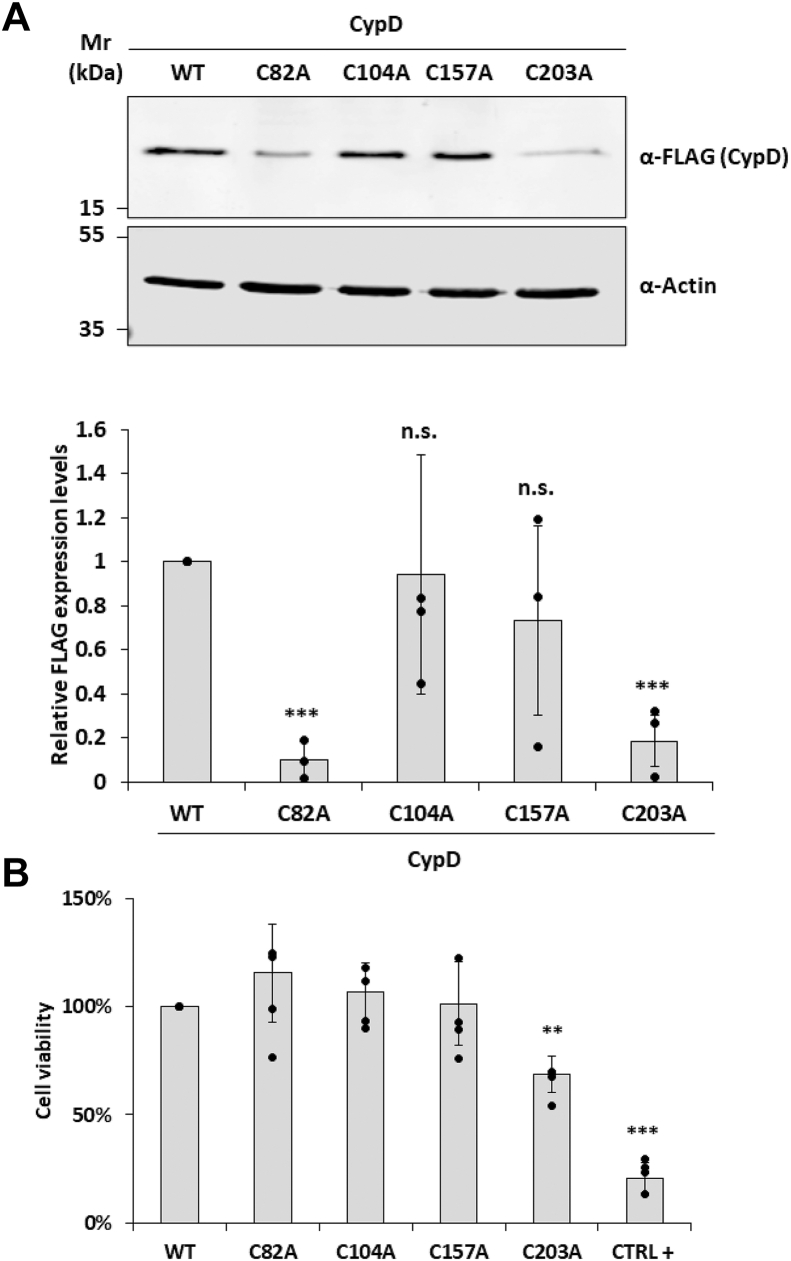
**Identification of cysteine residues involved in CypD/Mia40 disulfide bridge formation.***A*, Western blot analysis of the expression of CypD with mutated cysteines Cys82Ala, Cys104Ala, Cys157Ala, and Cys203Ala. Anti-FLAG antibody was used to visualize the expression of ectopic CypD and anti-Actin antibody for normalization of the samples. Both, CypD Cys82Ala and Cys203Ala mutants showed a significant reduced expression. Data reported are the mean ± SD of three biological replicates (∗∗∗: *p* ≤ 0.001). *B*, cell viability assay after transient transfection of CypD-WT or mutated cysteines Cys82Ala, Cys104Ala, Cys157Ala, and Cys203Ala. Cell viability was assessed 12 h post-transfection using MTS assay. CypD Cys203Ala mutant exhibited a significant loss of cell viability. H_2_O_2_-treated cells were used as positive control. Data reported are the mean ± SD of four biological replicates (∗∗: *p* ≤ 0.01, ∗∗∗: *p* ≤ 0.001).

To further characterize the molecular interaction between Mia40 CypD, we deepened our analysis by transiently expressing the single cysteine CypD point mutants in HeLa cells: Cys82Ala, Cys104Ala, Cys157Ala, and Cys203Ala. The expression levels of CypD-Cys104Ala and CypD-Cys157Ala were comparable with CypD WT, while the expression of both CypD-Cys82Ala and CypD-Cys203Ala was significantly lower (Cys82Ala: 10 ± 5%; Cys203Ala: 18 ± 12%) (Fig. 3*A*). To further investigate the biological effects related to the expression of CypD mutants, we conducted a cell viability assay using the tetrazolium salt MTS (3-(4,5-dimethylthiazol-2-yl)-5-(3-carboxymethoxyphenyl)-2-(4-sulfophenyl)-2H-tetrazolium). Tetrazolium salts such as MTS are reduced by mitochondrial and cytosolic dehydrogenase in metabolically active cells and represent, therefore, a functional readout of how mutations of CypD cysteines impact cell viability. As visible in Figure 3*C*, only cells expressing CypD-Cys203Ala show a reduced viability compared to the WT or the other point mutants. Treatment with hydrogen peroxide was used as a positive control of the assay (CTRL+).

### CypD mitochondrial localization is dependent on Mia40 expression

**Figure 4:**
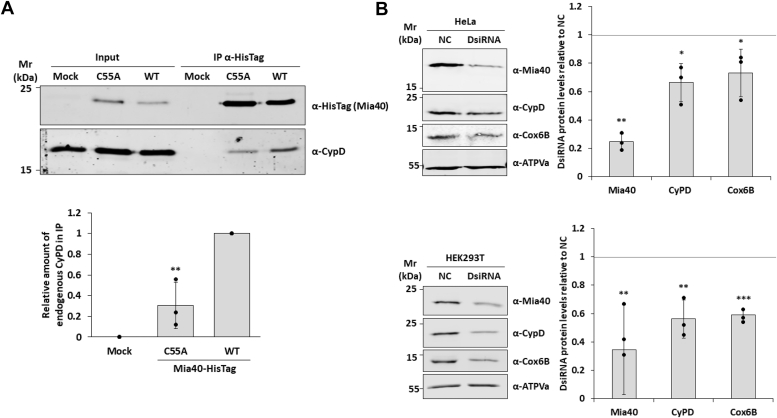

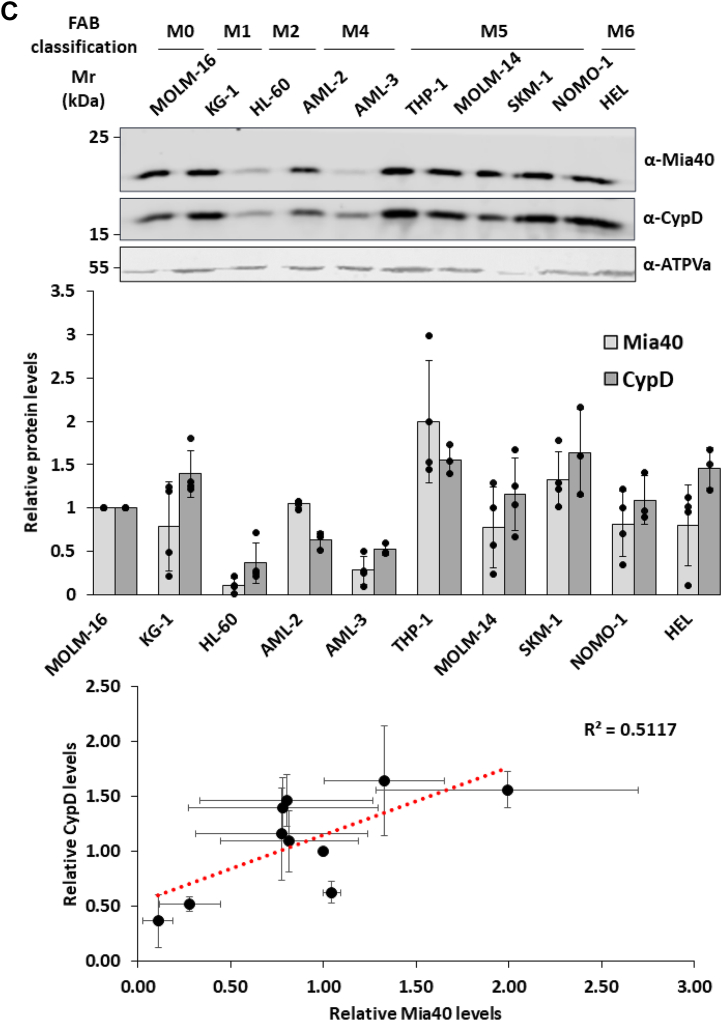
**CypD relies on the MIA pathway for the import in mitochondria.***A*, Western blot analysis of affinity purification from HeLa cells expressing Mia40-HisTag wild type (WT) and Cys55Ala mutant. HeLa cells were transiently transfected with pCMV5.1 (Mock) and pCMV5.1-Mia40-WT-HisTag or Mia40-Cys55Ala-HisTag vectors. Control (Mock) and IP samples were immunoprecipitated under native conditions from total HeLa cell extracts, separated by 12% SDS-PAGE, and analyzed by Western blot. Endogenous CypD is enriched in the IP fraction of the sample expressing recombinant Mia40-WT0-HisTag. Data reported are the mean ± SD of three biological replicates (∗∗: *p* ≤ 0.001). *B*, Western blot analysis of HeLa (*Top panel*) and HEK-293 (*bottom panel*) cell where the expression of Mia40 was reduced *via* specific siRNA expression. Mitochondrial protein extracts were isolated from cells transfected with oligonucleotides that targeted Mia40 mRNA (DsiRNA) or with control (NC) oligonucleotides. Reduction of Mia40 expression determined a decrease in CypD levels in mitochondria. Cox6B, a known Mia40 substrate, was used as positive control showing reduced levels in mitochondria. Data reported in the graphs are the mean ± SD of three biological replicates (∗: *p* ≤ 0.05, ∗∗: *p* ≤ 0.001, ∗∗∗: *p* ≤ 0.001). *C*, Western blot analysis of Mia40 and CypD expression levels in mitochondrial extract from 10 acute myeloid leukemia cell lines with different FAB classification (*upper panel*). ATPVa has been used as loading controls. In the graph (*central panel*) is reported the densitometry analysis of four independent biological replicates. Data reported are the mean ± SD of four biological replicates. Q–Q plot (*lower panel*) confirms the correlation between the expression of Mia40 and the mitochondrial levels of CypD.

A key step of the mitochondrial disulfide relay system is the formation of a mixed disulfide bond between Mia40 through its Cys55 and its substrate proteins (19). To further test the role of Mia40 in the translocation of CypD, Mia40-WT-HisTag (WT) or Mia40-Cys55Ala-HisTag (C55A) were transiently expressed in HeLa cells and immunopurified using an agarose bead-conjugated anti-HisTag antibody (Fig. 4*A*). Mia40-WT-HisTag showed a strong interaction with the endogenous CypD, while when the Cys55Ala mutant was expressed, this interaction was significantly reduced (31 ± 23%). Next, we examined the correlation of CypD and Mia40 expression levels in mitochondria. After silencing Mia40 in HeLa and HEK-293 cells, we saw a significant reduction in the amount of CypD (69 ± 15%) (Fig. 4*B*). To assess whether this observation was consistent in physiological conditions, we analyzed the expression patterns of CypD and Mia40 in isolated mitochondria from 10 different human leukemia cell lines (MOLM-16; KG-1; HL-60; OCI-AML2; OCI-AML3; THP-1; MOLM-14; SKM-1; NOMO-1; HEL). The representative Western blot analysis reported in Figure 4*C* shows that CypD and Mia40 follow a similar expression pattern, with higher amount of CypD in mitochondria of cell lines with higher expression of Mia40 and *vice versa*. The Shapiro–Wilk tests indicated that both variables, Mia40 and CypD, were normally distributed (*p* = 0.4349 and *p* = 0.4371, respectively). A Pearson correlation analysis revealed a strong positive correlation between Mia40 and CypD expression (r = 0.71), which was statistically significant (*p* = 0.020).

## Discussion

The mitochondrial import of nuclear-encoded proteins is a highly regulated process critical for maintaining mitochondrial function and cellular homeostasis. CypD is a conserved peptidyl-prolyl cis-trans isomerase localized in the mitochondrial matrix, where it performs crucial regulatory functions. It is best known as a central component of the MPTP, a non-specific channel whose opening can trigger necrosis and apoptosis in response to calcium overload, oxidative stress, or metabolic imbalance (6). Despite its physiological relevance, the mechanism by which CypD is imported into mitochondria has not been clearly defined.

Our study, providing multiple lines of evidence for an interaction between CypD and Mia40, identifies CypD as a novel non-canonical substrate of the MIA pathway. Since our purpose was to study the translocation of CypD from the cytoplasm into the mitochondria, unlike previous studies (17, 20, 21), we analyzed the full-length CypD protein containing the N-terminal pre-sequence, which is cleaved upon translocation into the mitochondrial matrix, creating an ∼19 kDa final product (10). We demonstrated that ectopically expressed FLAG-tagged CypD localizes exclusively to mitochondria, mirroring the localization of endogenous CypD. Moreover, *in organello* assays revealed that CypD mitochondrial internalization occurs independent of membrane potential.

Using complementary biochemical approaches, we showed that the interaction between Mia40 and CypD involves the formation of a disulfide bridge. In agreement with published data, mutation of Mia40 at Cys55 prevents this interaction. On the CypD side, mutation of either Cys104 or Cys157 did not affect protein expression, while mutation of Cys82 or Cys203 significantly reduced expression levels *in vivo*. These results suggest that the Cys104–Cys157 pair is not essential for folding and stability, making their involvement in Mia40 binding less likely. In contrast, expression levels of the Cys82 and Cys203 mutants were approximately 20% of WT levels, precluding co-immunoprecipitation analyses to assess Mia40 binding. Attempts to express these mutants *in vitro* yielded <10% of WT protein levels, preventing successful purification. These findings further support a critical role for this cysteine pair in CypD folding. Cell viability assays showed that expression of the Cys203Ala mutant significantly reduced viability compared to WT and other mutants. Based on these observations, we hypothesize that Mia40 Cys55 forms a transient disulfide bond with CypD Cys82, and that the substrate is subsequently released upon formation of an intramolecular disulfide bond between Cys82 and Cys203. Mutation of either residue may prevent Mia40 binding, thereby impairing CypD folding and promoting its degradation. However, while the Cys82 mutation appears to affect only CypD import, the Cys203 mutation exerts a more severe phenotype by potentially blocking the MIA pathway. Lacking the second cysteine necessary for substrate release, the Cys203 mutant may act as a “conformational anchor,” trapping Mia40 in a reduced state and preventing its reoxidation by ALR, thereby functionally inhibiting the entire MIA system. This could explain the pronounced reduction in cell viability observed upon Cys203 mutant expression. Unfortunately, technical limitations related to the poor expression of these mutants *in vitro* and *in vivo* prevented direct validation of this hypothesis.

Beyond expanding the known substrate repertoire of the MIA pathway, a key innovation of this study lies in the physiological implications of Mia40-dependent import of CypD. Given that CypD activity regulates MPTP and is itself modulated by oxidative stress, redox-dependent import may provide a mechanism to fine-tune mitochondrial susceptibility to permeability transition in response to ROS. This model would position Mia40 not only as an import factor but also as a redox-sensitive regulator of mitochondrial stress responses. Additionally, the variability in Mia40 expression across AML cell lines suggests that CypD import may be subject to tissue or context-specific regulation. For instance, leukemia-derived cell lines with low Mia40 levels showed reduced mitochondrial CypD content, suggesting that import efficiency may be modulated by the oxidative or metabolic state of the cell.

**Figure 5:**
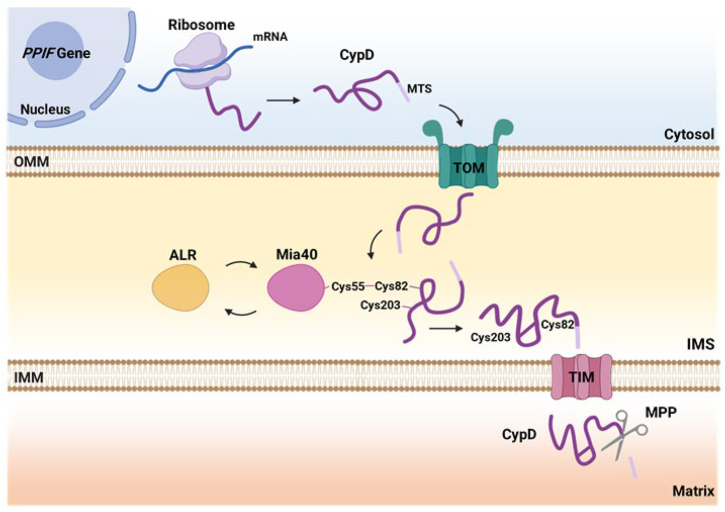
**Proposed model of CypD import pathway into mitochondria.** The figure illustrates the proposed import mechanisms of CypD into mitochondria *via* the Mia40-dependent oxidative folding system. Once in the IMS, CypD interacts with Mia40 Cysteine 55. Mia40 facilitates the import of CypD by forming disulfide bonds through CypD Cys82 and Cys203, which are critical for its correct folding and stabilization. Following this, CypD is translocated into the matrix where, according to the data reported in literature, undergoes the proteolytic removal of the MTS by a so far unidentified MPP.

In summary, our study identifies CypD as a novel non-canonical substrate of the Mia40-dependent oxidative folding pathway (Fig. 5). Our findings expand the functional scope of the MIA system beyond the IMS and propose a redox-regulated mechanism for CypD maturation and stability. Importantly, our results suggest that CypD import may be dynamically modulated by cellular redox status, providing a mechanistic link between oxidative stress, protein import, and mitochondrial permeability transition. This work broadens our understanding of mitochondrial proteostasis and offers new perspectives on how redox signaling influences mitochondrial function.

## Experimental procedures

### Cell culture and treatment

HeLa and HEK-293 cells were grown in Dulbecco’s modified Eagle’s medium (DMEM, Thermo Fisher) with 10% fetal bovine serum (FBS, Gibco). OCI-AML2, KG-1, HL-60, HEL and NOMO-1 cells were cultured in RPMI-1640 media (Gibco), supplemented with 10% FBS. MOLM-14, MOLM-16, THP-1 and SKM cells were grown in RPMI-1640 media supplemented with 20% FBS. OCI-AML2 and OCI-AML3 cells were grown in alpha-MEM (Gibco) with ribo- and deoxyribonucleosides supplemented with 10% FBS. All media were completed with 1 mM glutamine, 100 U/ml penicillin (Capricorn), and 10 μg/ml Streptomycin (Capricorn). HeLa and HEK-293 cell lines were available in our laboratory, while the AML cell lines were kindly provided by Dr Natalia Baran from the Institute of Hematology and Transfusion Medicine in Warsaw.

### Transient transfection experiments

HeLa cells were transiently transfected to express FLAG-CypD WT, FLAG-CypD mutants (Cys82Ala, Cys104Ala, Cys157Ala and Cys203Ala), HisTag-Mia40 WT and HisTag-Mia40 C55A mutant. One day before transfection, 8 × 10^4^, 3 × 10^5^ or 3 × 10^6^ cells were seeded in 24-well plates (with and without coverslips), 6-well plates or 10 cm^2^ plates, respectively. Cells were transfected with 800 ng, 6 μg or 12 μg of pCMV5.1 vector the day after using Lipofectamine 2000 reagent (Invitrogen). Cells were harvested 24 h after transfection.

### Preparation of total cell extracts

Total cell extracts were obtained from cells harvested by scraping followed by centrifugation at 650*g* for 5 min at 4 °C. After removing the supernatant, one wash was done with ice-cold phosphate-buffered saline 1X (PBS) and then centrifuged following the same conditions used before. The cell pellet was resuspended in cell lysis buffer [50 mM Tris-HCl (pH 7.5), 150 mM NaCl, 1 mM EDTA pH 8.8, 1% [wt/vol] Triton X-100] supplemented with 1X protease inhibitor cocktail (Thermo Fisher) and incubated at 4 °C for 30 min. Finally, samples were centrifuged at 12,000*g* for 20 min at 4 °C. Supernatant was collected and quantified using the Bradford assay.

### Preparation of subcellular fractionations

10 × 10^6^ cells were resuspended in 250 μl of Grinding solution [250 mM Sucrose, 2 mM EDTA (pH 7.4)] supplemented with BSA 0.1%. HeLa cells were sonicated for 4 s to obtain intact nuclei. Following a 20 min centrifugation at 650*g* at 4 °C, the supernatant was processed for mitochondria isolation, while the pellet was collected to lyse the nuclear fraction. The supernatant was centrifuged at 16,000*g* for 20 min at 4 °C. The obtained pellet was washed twice in Grinding solution without BSA and centrifuged at 16,000*g* for 10 min at 4 °C. The final pellet was resuspended in Grinding solution and quantified using the Bradford colorimetric assay. For the nuclear fraction preparation, the pellet was washed twice in 400 μl of T1 solution [10 mM Hepes (pH 7.9), 10 mM KCl, 0.1 mM MgCl2, 0.1 mM EDTA (pH 8)] supplemented with protease inhibitor and 2 mM PMSF, and centrifuged at 650*g* for 15 min at 4 °C. The resulting pellet was resuspended in Lysis solution T2 [20 mM Hepes (pH 7.9), 420 mM NaCl, 1.5 mM MgCl2, 0.1 mM EDTA (pH 8), 5% Glycerol] supplemented with 0.01% Triton X-100, incubated on ice for 20 min, and centrifuged at 18,000*g* for 20 min at 4 °C. Supernatant was collected as the nuclear fraction and quantified *via* Bradford analysis.

### Western blot analysis

The indicated amounts of total, nuclear or mitochondrial extracts were loaded into a 12% SDS-PAGE unless otherwise specified. After transfer, membranes were blocked by incubation with 5% non-fat dry milk resuspended in TBS 1X- 0.1% Tween20 for 1 h at room temperature. The following primary antibodies were used overnight at 4 °C in agitation: anti-ATPVa monoclonal 1:1000 (ab14748, Abcam), anti-LSD1 monoclonal 1:1000 (EPR6825, Proteintech), anti-CypD monoclonal 1:1000 (18466-1-AP, Proteintech), anti-MIA40 1:1000 polyclonal (21090-1-AP, Proteintech), anti-GST 1:1000 monoclonal (ab18183, Abcam), anti-HisTag monoclonal (MA1-21315, ThermoScientific) and polyclonal (10001-0-AP, Proteintech), anti-FLAG monoclonal 1:1000 (F3165, Sigma Aldrich), and Cox6B 1:250 monoclonal (ab110266, Abcam). The specificity of the antibodies is guaranteed by the company and by the migration on SDS-PAGE based on molecular weight. The day after, membranes were washed three times with TBS 1X-0.1% Tween 20 and incubated for 2 h with the secondary antibody. Signals were detected with Odyssey DLx Scanner (Li-Cor Biosciences) and quantified by using ImageStudio software (Li-Cor Biosciences).

### Immunofluorescence

8 × 10^4^ cells were seeded on a glass coverslip. Cells were washed 3 times with 1X PBS, fixed for 20 min with 4% (w/v) paraformaldehyde, washed again 3 times with 1X PBS and permeabilized for 5 min with 0.5% Triton X-100 in 1X PBS. Blocking was performed in 10% goat serum in 1X PBS for 1 h. The following primary antibodies were incubated overnight at 4 °C: anti-FLAG 1:100 monoclonal (F3165, Sigma Aldrich); anti-CypD 1:100 monoclonal (18466-1-AP, Proteintech); anti-ATPVa monoclonal 1:250 (ab14748, Abcam); anti-TOMM20 monoclonal 1:250 (MA5-55630, Thermo Fisher)). All antibodies used are commercially available and tested for IF analysis. The day after, cells were washed 3 times with solution A (Duolink) for 5 min and incubated with the reported Alexa Fluor fluorophore secondary antibody (ThermoFisher Scientific) for 2 h at room temperature. Nuclei were stained with Hoechst 33342. For that, cells were washed 2 times with solution A (Duolink), afterwards Hoechst 33342 (1:10,000 in 1X PBS) was added to the cells for 5 min in agitation. Afterward, cells were washed once with solution A, 2 times with solution B (Duolink) and one time with solution B 1:100 in 1X PBS. The acquisition of the images was performed using an inverted Leica TCS SP8 Confocal Laser Scanning Microscope (Leica Microsystem) with a White Light Laser (WLL) or with the LSM 800 Airyscan (Zeiss) for super-resolution images, both with 12-bit depth and 1024 × 1024 pixel resolution.

### DsiRNA

The day before the experiment 1.5 × 10^5^ cells per well were seeded in 6-well plates. Two wells of a 6-well plate were used for each condition. For each well transfection, 150 μl of OptiMEM medium was mixed with 1.5 μl of siRNA (For: 5′-UUGAGGCCACUGCAACCAAAGAAGA-3′; Rev: 5′-UCUUCUUUGGUUGCAGUGGCCUCAAUG-3′; IDT Reference #510052170) or control siRNA (DS NC1; IDT Reference # 51011403). In parallel, 150 μl of OptiMEM medium was mixed with 9 μl of RNAiMAX Lipofectamine (Invitrogen). The two reactions were mixed together and incubated at room temperature for 5 min to allow for complex formation. After incubation, 250 μl of the prepared transfection mixture was added to each well containing 2 ml of growth medium without antibiotics. Cells were then incubated for 48 h (HeLa) and 72 h (HEK-293) at 37 °C in a 5% CO_2_.

### Expression and purification of recombinant proteins

Recombinant CypD-His was synthesized using RTS 100 Wheat Germ Kit (BiotechRabbit) following the manufacturer’s instructions. Briefly, a feeding solution was prepared using 900 μl of Feeding solution, 80 μl of amino acids and 20 μl of methionine. Reaction solution was prepared using 15 μl of Reaction Mix, 4 μl of amino acids, 1 μl of methionine, 15 μl of Wheat Germ Extract and 3 μg of the corresponding plasmid. Each of the solutions was added to the corresponding compartment in the synthesis chamber and incubated for 24 h at 24 °C and 900 rpm. The produced protein was purified using 20 μl Ni-NTA agarose QIAGEN beads. The mixture was incubated at 4 °C for 30 min in rotation. Following incubation, the tube was centrifuged at 1000*g* for 10 s to pellet the resin, and the supernatant containing unbound proteins was carefully transferred to a fresh tube and labelled as flow-through (FT) fraction. The resin was subsequently washed twice using 100 μl of wash buffer per wash. 20 μl of elution buffer were added to the resin and followed by rotation and incubation at room temperature for 10 min. The tube was then centrifuged at 1000*g* for 10 s, and the eluted protein was transferred to a fresh tube. The elution step was repeated twice. Recombinant GST and GST-Mia40 were synthesized in *E.coli* and purified using GSTrap HP columns as previously described (15).

### *In organello* import analysis

Freshly isolated mitochondria (30 μg) from HEK-293 cells were resuspended in 30 μl of import buffer [250 mM sucrose, 80 mM potassium acetate, 5 mM magnesium acetate, 10 mM sodium succinate, 20 mM HEPES/KOH, pH 7.4] and incubated at 30 °C for 2 min. 5% (v/v) of *in vitro* synthesized CypD was added to the reaction and the mixture was incubated at 30 °C for 30 min. When required, valinomycin (0.1 mM), oligomycin (1 mM), and antimycin (0.8 mM) were added. Following proteinase K digestion with 25 μg/ml for 15 min on ice, PMSF was added to a final concentration of 2.5 mM, and the reaction was incubated on ice for 5 min. Samples were centrifuged at 20,000*g* for 10 min at 4 °C. The supernatant was removed, and the pellet was washed once with 100 μl of HS buffer [500 mM sucrose, 20 mM HEPES/KOH, pH 7.4]. Samples were centrifuged again at 20,000*g* for 10 min at 4 °C, and the pellet was resuspended in 1X Laemmli buffer.

### GST pull-down assay

15 pmol of GST-Mia40-HisTag were incubated with 30 pmol of recombinant CypD-HisTag in 100 μl of binding buffer [50 mM Tris-HCl pH 8.0, 10% glycerol, 0.5% NP-40] for 3 h under rotation at 4 °C. 10 μl of glutathione-Sepharose 4B beads (GE Healthcare) were added to the mixture for 30 min under rotation at 4 °C. Before elution, samples were washed three times with wash buffer [50 mM Tris-HCl pH 8.0, 1% NP-40, PBS 1X]. Elution was performed with 50 μl of the elution buffer (50 mM Tris-HCl pH 8.0 in 1X PBS, with freshly added 10 mM GSH). 25 μl of each sample were separated on 12% SDS-PAGE gel using reducing Laemmli 1X [250-mM Tris-HCl pH 6.8, 8% SDS, 40% Glycerol, 0.08% Bromophenol Blue, 20% β-mercaptoethanol] or non-reducing Laemmli 1X [250-mM Tris-HCl pH 6.8, 8% SDS, 40%, Glycerol, 0.08% Bromophenol Blue] and Western blot analysis was performed using the reported primary antibodies.

### FLAG-Tag affinity purification

Total cell extracts were obtained as described in preparation of the total cellular extracts section using 800 μl of lysis buffer and incubated for 3 h at 4 °C with 45 μl of anti-FLAG M2 affinity gel resin (Sigma Aldrich) pre-washed 3 times with ice-cold 1X TBS. After the incubation beads were washed three times with 1X TBS. Proteins were eluted by incubation for 45 min with 0.15 mg/ml 3xFLAG peptide in 1X TBS.

### HisTag affinity purification

6 × 10^6^ cell were collected and lysed as described above using Co-IP Lysis Buffer [5% glycerol, 20 mM Tris-HCl pH 7.4, 150 mM NaCl, 1 mM EDTA, 0.5% NP-40, 1.5 mM MgCl_2_, and 1X Protease Inhibitor). Anti-His antibody (Invitrogen) was added to the lysate and incubated for 4 h at 4 °C under rotation. Agarose beads (Sigma Aldrich) were washed three times with cold 1X PBS, and the antibody-bound lysate was then added to the beads and incubated for 1 h at 4 °C under rotation. Following three washes in 1X PBS, elution was performed using 50 μl of 1X Laemmli buffer, incubating the reaction at 95 °C for 10 min. Finally, samples were centrifuged at 3000*g* for 15 min. Supernatant was collected, and 25 μl were loaded on a 12% SDS-PAGE gel.

### Proximity ligation assay

8 × 10^4^ cells were seeded on a glass coverslip. The day after, cells were transiently transfected with FLAG-tagged form of CypD-WT, C82A, C104A, C157A or C203A mutant. 12 h after transfection cells were fixed with 4% (w/v) paraformaldehyde in PBS 1X for 20 min, permeabilized with Triton X-100 0.25% in PBS 1X for 5 min and blocked with goat serum 10% in PBS 1X for 1 h. Primary monoclonal antibody anti-FLAG (Sigma Aldrich) was incubated for 3 h at 37 °C at a final dilution of 1:1000. After washing three times with TBS 1X - 0.1% Tween-20, cells were incubated with the primary monoclonal antibody anti-Mia40 (21090-1-AP, Proteintech) diluted 1:1,200, overnight at 4 °C. PLA was conducted following the manufacturer's instructions. PLA mouse and rabbit probes (Duolink) were diluted 1:5. Ligation solution was diluted 1:5 in water and 1 μl of ligase was added to the solution and incubated on the coverslip for 30 min at 37 °C. Samples were washed for 2 min twice using wash Buffer A, and an amplification reaction was conducted by diluting the amplification stock solution 1:5 in water and adding 0.5 μl of polymerase for 100 min at 37 °C. Nuclei were stained with Hoechst 33,342. Images were analyzed using an inverted SP8 microscope (Leica Microsystems) with a White Light Laser (WLL).

### Cell viability assay

8 × 10^4^ HeLa cells were seeded in 96-well plates and transfected with either pCMV-CypD-WT or one of the cysteine mutants (Cys82Ala, Cys104Ala, Cys157Ala, and Cys203Ala). 12 h after transfection, cell viability was assessed using CellTiter 96 AQueous One Solution Cell Proliferation Assay (Promega), according to the manufacturer's instructions. Briefly, 20 μl of CellTiter reagent were added to each well containing cells in 100 μl of DMEM medium. Plates were incubated at 37 °C for 4 h, and absorbance was measured at 490 nm using a microplate reader (Synergy H1 Neo Plate Reader, Biotek). As positive cell death control, HeLa cells were treated with 250 μM H_2_O_2_ for 4 h.

### Statistical and bioinformatic analysis

Statistical analysis was performed using Microsoft Excel. One-way ANOVA was used for three group comparisons and Student’s *t*-test was used for two group comparisons. *p* values of less than 0.05 were considered significant, while values less than 0.01 or lower were considered highly significant. For correlation analysis of Mia40 and CypD in AML cell lines, normality of the datasets was assessed using the Shapiro–Wilk test. Since both variables were normally distributed (*p* > 0.05), a Pearson correlation test was performed. 3D structures of the proteins were retrieved from the Protein Data Bank and analyzed or modified using ChimeraX UCSF.

## Data availability

All data are contained in the manuscript. Raw data are available on Zenodo (https://doi.org/10.5281/zenodo.17043888). Plasmids and specific reagents will be made available upon request to the corresponding author.

## Supporting information

This article contains supporting information.

## Conflict of interest

The authors declare that they have no conflicts of interest with the contents of this article.

## Supporting information

Supplementary informations
