# Supplementary material for: Cyclophilin D is a new non-canonical substrate of the mitochondrial intermembrane space assembly pathway
Source: J Biol Chem. 2025 Nov 4;301(12):110883. doi: 10.1016/j.jbc.2025.110883 (PMC12732308; doi:10.1016/j.jbc.2025.110883)
Supplement: Supplementary informations [file mmc1.docx]

**Cyclophilin D is a new non-canonical substrate of the mitochondrial intermembrane space assembly pathway**

Mara Equisoain Redin^1,2#^, Veronica Bazzani^1#^, Eve Harding^1^, Joshua McHale^1,2^, and Carlo Vascotto^1,2*^

^1^IMol Polish Academy of Sciences, 02-247 Warsaw, Poland.

^2^Department of Medicine, University of Udine, 33100 Udine, Italy.

* To whom correspondence should be addressed. Tel: +39.0432.494310; Email: carlo.vascotto@uniud.it

# These authors have contributed equally to the present work.


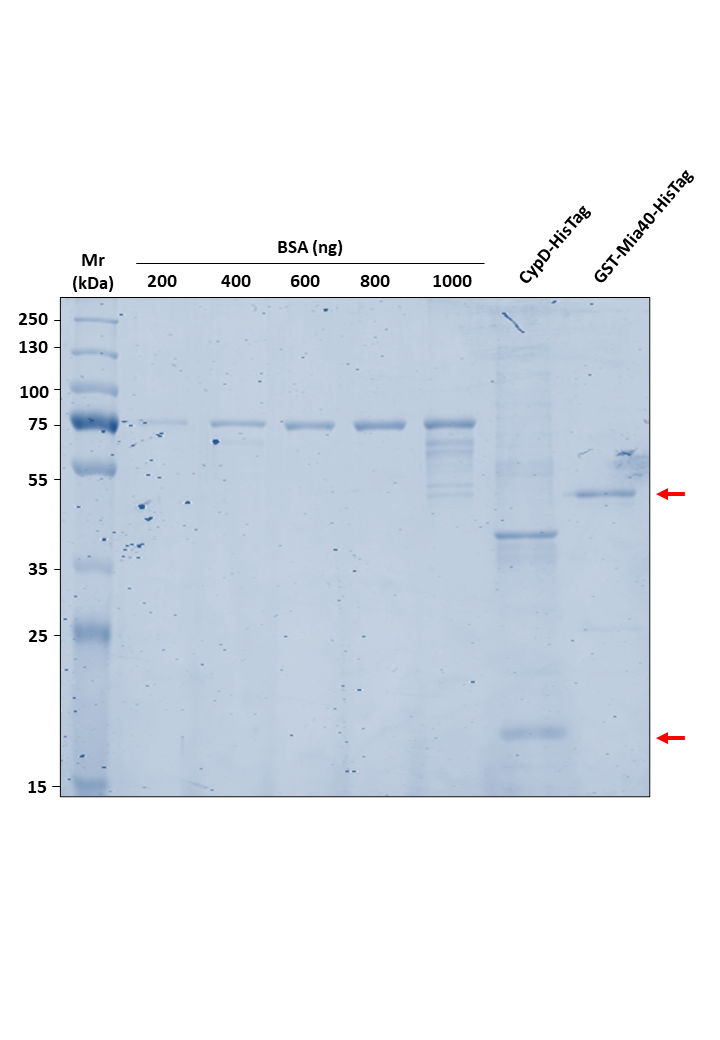


**Supplementary Figure 1: SDS-PAGE analysis of recombinant CypD and GST-Mia40-HisTag.** Recombinant CypD and GST-Mia40-HisTag were expressed and purified as described in the Materials and Methods section and quantified using the Bradford assay. A total of 500 ng of each recombinant protein was separated by SDS-PAGE. Reported amounts of BSA were loaded in parallel to generate a standard curve for accurate quantification of the recombinant proteins. The gel was stained with Coomassie Blue, and densitometric analysis was performed using a Typhoon laser scanner following image digitization.
